# Use of Cryopreserved Human Umbilical Cord for Wound Healing of the Nose after Mohs Micrographic Surgery

**DOI:** 10.1155/2022/2107629

**Published:** 2022-03-08

**Authors:** Kathleen Herne, Robert K. Fabric

**Affiliations:** ^1^Integrated Dermatology of West Palm Beach, West Palm Beach, FL, USA; ^2^Department of Surgery in the Division of Plastic Surgery at the Leonard M. Miller School of Medicine, Miami, FL, USA

## Abstract

Mohs micrographic surgery (MMS) is microscopically controlled surgery used to treat common skin cancers including primary and recurrent basal cell carcinoma (BCC). Unfortunately, postexcisional MMS wounds, particularly down to periosteum or perichondrium, are susceptible to potentially poor cosmetic outcomes, including wound contracture, hypopigmentation and hyperpigmentation, and contour mismatch. Herein, we report a case to show how adjunctive application of human cryopreserved umbilical cord tissue (UC) may expedite wound healing with improved aesthetic outcome. A 53-year-old Caucasian female with a slight natural lifelong depression between her lower nasal tip alar cartilages suffered from a recurrent BCC mostly of the right nasal tip. After MMS down to the perichondrium, UC was immediately applied to the surgical wound. Prolific granulation developed at one week followed by rapid reepithelialization at two weeks. This resulted in complete closure at four weeks and a pleasing aesthetic nasal tip at 6 weeks. At one year and 5 months after MMS, there continued to be excellent aesthetic outcome as evaluated by surrounding skin color, contour, and texture with a minimal residual scar. In this case, the adjunctive use of cryopreserved human UC accelerated the postsurgical MMS wound healing in the nasal tip particularly in patients with significant comorbidities or are unwilling to undergo a formal surgical reconstruction. This encouraging finding warrants further controlled studies in the future.

## 1. Introduction

Basal cell carcinoma (BCC) is the most common skin malignancy with an increasing incidence worldwide [1]. The disease primarily affects older Caucasian populations and often appears on the face. Treatment options include topical therapies (fluorouracil and imiquimod), photodynamic therapy, laser treatment, radiation, and surgery [1]. Mohs micrographic surgery (MMS) offers high cure rates with maximal normal tissue preservation in sites at risk for high rates of recurrence. Post-MMS wounds can be managed by primary closure if the wound is small, but if the wound is more extensive, it may require reconstructive surgery with flaps (local or distant) or skin grafts (split or full thickness). Nonetheless, wound healing can be complicated by contracture (including a trapdoor deformity), hypopigmentation, hyperpigmentation, contour mismatch to the surrounding skin, and unsightly scarring of both the surgical and donor sites. The umbilical cord (UC) contains the same histological features as amniotic membrane except that the avascular stroma contains more hyaluronic acid and was selected as an adjunctive therapy for this patient because it had been demonstrated in various types of wounds [2, 3] to elicit anti-inflammatory, antiscarring, and regenerative healing properties.

## 2. Case Presentation

**Figure 1:**
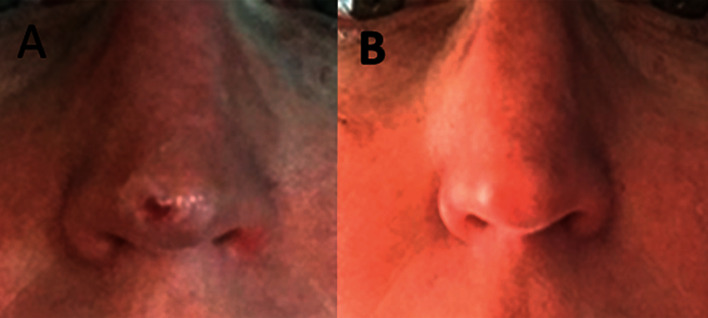
Three years prior to the present illness, the primary lesion of the right nasal tip was confirmed as BCC (a) through a shave biopsy on 1/24/2017. Approximately a month later in February (b), the wound was healed, and the patient was started on a full course of imiquimod.

**Figure 2:**
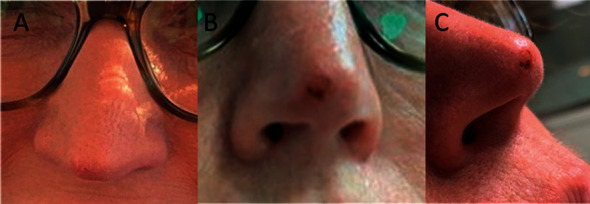
Three years later, the patient presented with a recurrent lesion on the nasal tip on 12/3/2019 (a) confirmed as recurrent BCC following a shave biopsy (b and c).

Three years prior, a 53-year-old Caucasian female presented with a nonhealing lesion on her nose of several months' duration (Figure 1(a)). A shave biopsy revealed BCC, and the site healed within one month (Figure 1(b)). At that time, due to the patient's use of blood thinners for pulmonary emboli, extensive surgical intervention was not indicated; therefore, the patient was placed on a full course of topical imiquimod (Aldara®; Bausch Health, Bridgewater Township, NJ). During this three-year interim, the patient was doing well until a recent slowly enlarging papule appeared at the same site (Figure 2(a)). The patient underwent a shave biopsy (Figures 2(b) and 2(c)) that confirmed the lesion as a recurrent BCC of the nodular infiltrative type.

**Figure 3:**
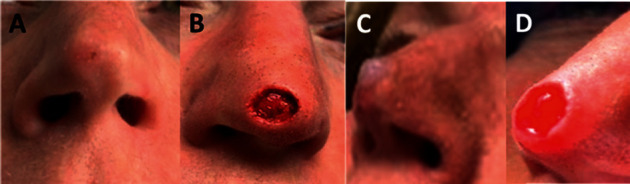
The patient underwent MMS down to the perichondrium on 2/27/2020 ((a) before and (b) immediately after MMS). UC was immediately applied (c), and then, a new piece of UC allograft with nonadherent dressing was applied weekly for the following three weeks with minimal debridement. Prolific granulation tissue was formed by one week, on 3/5/2020 (d).

**Figure 4:**
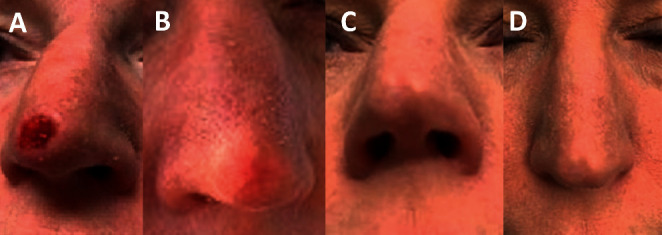
Evident peripheral epithelization from around the edge by two weeks (a); after a final UC graft was applied, the wound was fully reepithelialized and closed at four weeks (b). At one year and 5 months after MMS with UC (c, d) the wound is fully matured revealing a soft nasal tip with matched contour, color, and texture to the surrounding area and maintained the normal nasal tip light reflex.

The patient was advised to undergo MMS. Preoperatively, she consulted with a different plastic surgeon regarding her surgical repair, and his surgical plan depending upon the margins was to use either a skin graft or flap to reconstruct her nasal tip. Rather, based on the reported anti-inflammatory, antiscarring, and regenerative healing properties of UC, the patient was advised and consented with a plan that included MMS followed by the adjunctive use of UC. On the date of the Mohs surgery (Figure 3(a)), two stages were required to obtain tumor-free margins with the resultant wound size of 1.0 × 1.1 cm which crossed the midline of the nasal tip exposing the perichondrium of the nasal alar cartilage (Figure 3(b)). Immediately after MMS (Figure 3(c)), a UC allograft (NEOX CORD® 1K; Amniox Medical, Miami, FL) was cut to wound size and applied to the wound under a nonadherent moist dressing (Xeroform®; Covidien, Dublin, Ireland), cotton bolster, and adhesive tape (Omnifix, Hartmann USA, Rock Hill). The patient was instructed to leave the dressing in place until each follow-up office visit. By one-week after MMS, the wound displayed prolific high-quality granulation tissue with minimal inflammatory response (Figure 3(d)) without any evidence of infection. Rapid reepithelialization was evident at two weeks (Figure 4(a)), resulting in complete closure at four weeks (Figure 4(b)) and a pleasing aesthetic result in the nasal tip at 6 weeks. It should be noted in the literature that others have left the UC allograft in place for up to 3 weeks. However, in this case, the graft was reapplied with a new UC (NEOX CORD® 1K) allograft during weekly dressing changes of the 4 weeks of wound healing. At one year and 5 months after MMS, there was excellent aesthetic outcome as evaluated by surrounding skin color, contour, and texture with a minimal residual scar (Figures 4(c) and 4(d)).

## 3. Discussion

This case report highlights a successful aesthetic outcome using cryopreserved UC to accelerate post-MMS wound healing after a sizeable excision down to the perichondrium for a recurrent BCC of the nasal tip. The wound was fully closed within 4 weeks after MMS without any additional surgical intervention. Comparatively, typical healing times for secondary intention of similar wounds could range from 2 to 3 times as long, dependent upon the wound depth, size, and metabolic health of the patient [4]. This adjunctive weekly application of UC negated additional reconstruction, which carries an inherent risk of scarring including a trapdoor deformity, pigment irregularities, telangiectasias, texture and contour mismatch, and scarring associated with donor tissue for flaps or grafts. Similarly, the convexity of the distal third of the nose makes it a less-than-ideal area for quality healing by secondary intention. These wound problems are particularly undesirable on the face especially involving the nose which may generate future aesthetic concerns with its associated emotional overlay.

The UC used herein is a cryopreserved product that preserves hyaluronic acid (HA)-rich extracellular matrix and growth factors/cytokines therein but devitalizes all living cells [2, 5]. It is stored at 4^o^C to −80°C ready for immediate usage without rehydration. This UC allograft was used in preference of any other type of natal membranes because it is about ten times thicker and contains higher concentrations of biological components than pure amniotic tissue. Furthermore, such UC tissue has successfully been used to heal complex diabetic foot ulcers with evidence of exposed bone, tendon, muscle and/or joint capsule, orthopedic joint problems, spina bifida, radiation-induced wounds, and ischemic wounds [6–11]. The biological effects of UC have been attributed to a unique extracellular matrix component known as Heavy Chain Hyaluronic Acid/Pentraxin-3 complex (HC-HA/PTX3), which modulates the local inflammatory environment by inducing apoptosis of activated inflammatory cells and promotes an antiscarring effect by downregulating transforming growth factor (TGF)-*β*, preventing myofibroblast formation [3]. These regenerative biological actions are in contrast to properties of other available xenografts such as acellular dermis which is primarily used as a mechanical scaffolding that provides a structure for host cell migration and proliferation. This was verified in a head-to-head study, wherein UC was demonstrated to significantly reduce inflammation, reduce cell death, and promote superior cellular organization compared to acellular dermal matrix [12]. The safety of UC has also been demonstrated based on over 65,000 units distributed (per manufacturer) and shown not to increase recurrence after ocular, oral, dermal, and prostate tumor excision. Hence, UC was chosen to use in this case and reduce the known potential risks of conventional treatment including contracture, altered pigmentation, and contour mismatch. Overall, these properties collectively might explain why the adjunctive use of cryopreserved human UC accelerates post-MMS wound healing in the nasal tip particularly in patients with significant comorbidities or are unwilling to undergo a formal surgical reconstruction. This encouraging finding warrants further controlled studies in the future.

## Data Availability

All available data are included in this report.
